# Experiences of work ability in young workers: an exploratory interview study

**DOI:** 10.1007/s00420-015-1101-7

**Published:** 2015-10-29

**Authors:** Maria Boström, Kristina Holmgren, Judith K. Sluiter, Mats Hagberg, Anna Grimby-Ekman

**Affiliations:** Occupational and Environmental Medicine, Department of Public Health and Community Medicine, Sahlgrenska Academy, Sahlgrenska University Hospital, University of Gothenburg, Box 414, 405 30 Gothenburg, Sweden; Social Medicine, Department of Public Health and Community Medicine, Sahlgrenska Academy, Sahlgrenska University Hospital, University of Gothenburg, Gothenburg, Sweden; Academic Medical Center, Coronel Institute of Occupational Health, Amsterdam, The Netherlands

**Keywords:** Work ability, Qualitative interviews, Young workers, Alertness, Meaningfulness, Work climate, Work organization, Private life

## Abstract

**Purpose:**

The aim of this study was to explore the experiences of and influences on work ability in young workers related to their work and life situation.

**Methods:**

In a qualitative study of a strategic sample of 12 young female and 12 young male workers, aged 25–30 years, in work or recently left work, recruited from the 5-year follow-up of a Swedish cohort, semi-structured interviews were performed to explore the experiences of work ability in these young workers. Systematic text condensation inspired by phenomenology was used in the analysis.

**Results:**

Work ability was experienced as complex, consisting of four themes, each with three subthemes. To be alert and have energy, to possess sufficient education, skills and working life experience and experience meaningfulness and engagement in work, were perceived to be fundamental for work ability and were seen as the worker’s own responsibility. Moreover, work ability can be improved or reduced by the psychosocial work climate, the work organization and the private life. Optimal work ability was experienced when all themes integrated in a positive way.

**Conclusions:**

Work ability was experienced as the worker’s own responsibility that could be influenced by work circumstances and private life. To promote good work ability among young workers, work ability has to be understood in its specific context. Whether the understanding of work ability found in this study is explicit for the group of young adults needs to be explored in a more general population in further research.

## Introduction

Due to an aging population, a workforce with sustainable work ability is important. Young adults who are entering their working life today can expect to have a longer working life than that of the preceding generation and therefore need good work ability over many years. As well, young adults need special attention to ensure their work ability as they are new in the workplace, are often in the minority and can meet high expectations from the employer (Ilmarinen 2009).

The concept of work ability is complex as it has three dimensions: physical, psychological and social (Ludvigsson et al. 2006). There is no general definition of the concept, and it can vary between different fields such as medicine and law. In occupational health, the concept usually refers to the balance between an individual’s resources (such as health, knowledge and attitudes) and the working conditions (such as work content, work demands, and work organization) (Ilmarinen 2009). Work ability should be seen in the context of a close relationship to family, friends, the nearby environment and society.

Today, there is a lack of knowledge about what work ability may mean for young adults, since most studies are on mainly adult workers (Boschman et al. 2014; Leijten et al. 2014; Stigmar et al. 2010, 2012; van den Berg et al. 2009). However, work ability in young workers may be dependent on their interpretation and idea of work (Seitsamo et al. 2008). Only a few studies concern young adults. One such study is a Finnish quantitative cross-sectional study (Seitsamo et al. 2008) of 1500 young adults, 18–29 years old. The authors found that a poor basic education, high physical work demands and mental strain at work were related with poor work ability as measured by the work ability index (WAI). On the other hand, satisfaction with one’s health, good perceived quality of life, good physical fitness, male gender, young age and experiences of appreciation at work were shown to be the most important factors, in this sequence, related to the young adults’ excellent work ability. Thus, both work and life outside work appear to influence the perceived work ability of young employed people. In another Finnish study (Pohjonen 2001) that used the same measure of work ability, health status and also control and ergonomic factors at work predicted the level of work ability in female home care workers aged 19–34 years.

In a previously published quantitative study of the work situation in young workers (Bostrom et al. 2012), the authors found that changes in job control and changes in the negative influence of job demands on private life were the most important work factors associated with both reduced and improved work ability over time. The study was limited to examining the factors in depth using a questionnaire. Furthermore, the study investigated changes in factors in the work situation only and did not include individual and private life aspects.

Apart from one study of work ability in young adults conducted using structured interviews (Seitsamo et al. 2008), no research, to our knowledge, has investigated young workers’ own experience of work ability in a more detailed way. As it is plausible to think that a young group of workers could have another approach to work than adult workers (Cartwright and Holmes 2006), it is of importance to understand how young workers experience work ability. Also, a better understanding of work ability in this group could be the basis for developing improvements programs at the workplace. Currently, such programs are likely derived from knowledge about older workers. In addition, a phenomenological approach, like the one used in this study, that entails a few broad questions, offers the possibility of gaining new and interesting knowledge about work ability from the young workers themselves.

The aim of this study was to explore the experiences of and influences on work ability in young workers related to their work and life situation.

## Methods

A qualitative phenomenological approach was used for this study and systematic text condensation (STC) was selected as a relevant strategy (Malterud 2012). To obtain rich information on the experiences of work ability in young adults’ daily work, the data were collected through individual semi-structured interviews addressing four topics. The design of the study was developed by all authors and was largely performed by two of the authors (MB, KH), of whom the latter (KH) has experience in qualitative methodology. The first author is experienced in clinical work as a physiotherapist and also in occupational environmental work as an ergonomist. This experience can be seen as the pre-understanding that needed to be bridled (Dahlberg et al. 2008) throughout the research process. An explanation of the concept and the need for bridling follows.

### Theoretical frame

Phenomenology is based on theories of humans’ experiences (Giorgi 2008; Malterud 2009). To have a phenomenological approach in research is to look at objects, or phenomena, from the perspective of how they are experienced (Malterud 2012). One way to understand humans is to explore the meanings of their experiences in daily life, preferably using few interview topics instead of many predestinated questions, to obtain deep and rich data. To reach this understanding, an open mind is essential, one that does not contain the answer in advance. Bridling, which can be described as an individual keeping back what he or she believes is true and reflecting on what he or she takes for granted (Dahlberg et al. 2008), can be an useful attitude to maintain throughout the research process.

The selected strategy, STC, is based on Giorgi’s psychological phenomenology (Giorgi 2008), but with modifications (Malterud 2012). It entails a descriptive approach that reveals the participants’ own experiences as expressed by them and condensed into meanings.

### Sample and material

The 24 participants were selected from an ongoing population cohort of young adults, in which data were collected on a 5-year follow-up during 2012 and 2013 with 2738 responses. From this follow-up, we strategically selected a study sample of 265 individuals based on three criteria: (1) living in the region of Västra Götaland, Sweden, (2) in ongoing work or recently having left work and (3) having reported poor or excellent work ability. As the chosen participants were 25–30 years old, they were older than the World Health Organization (WHO) definition of young adult as aged 20–24 years. However, some researchers have suggested extending the definition of a young adult to 30 years of age (Trondman 2003).

To obtain a rich description of experiences (Malterud 2001) in young workers, eight subgroups were created from the study sample that differed by sex (women and men), education level (9 years of school or high school, respectively, college or university) and work ability level.

Work ability was measured by the work ability score (WAS) in the work ability index (WAI) (El Fassi et al. 2013; Gould et al. 2008) reported in the questionnaire. This single item for scoring work ability measures “current work ability compared with the lifetime best” and consists of a scale from 0, representing “cannot work at all right now,” to 10, representing “my work ability is at its best right now.” Poor work ability was defined as a self-reported WAS of ≤5. Nevertheless, for two of the eight subgroups it was necessary to use the definition of poor work ability as a self-assessed WAS of ≤6 to obtain enough participants to call for the interviews. This higher limit was still in line with the definition of poor work ability as a WAS ranging from 0 to 7, which had been used previously (Gould et al. 2008). Excellent work ability was defined as a self-reported WAS of 10 (Gould et al. 2008).

A total of 20–25 interviews were considered sufficient to draw valid conclusions (Dahlberg et al. 2008) from the collected data. While forming the subgroups, a number of 24 participants were decided to be suitable and these were randomly selected from the eight subgroups. The first three participants randomized in each subgroup received an invitation letter and then an inquiry by telephone 1–3 weeks later about possible participation. If anyone declined to participate or failed to appear at the agreed time, an additional selection of participants was made in the same manner as before. In total, 64 letters were sent out and one to three telephone calls per person was made. Of these invitations, 24 individuals were unreachable and 13 individuals declined to participate due to a lack of interest or lack of time. In total, 27 individuals who were contacted agreed to an interview: Three of them failed to appear for the interview and 24 individuals completed the interview. Of these 24 participants, 21 were in ongoing work, one had recently left work for study and two had been on sick leave for no more than 3 months when called for the interview.

The characteristics of the participants are shown in Table 1. Among the female employees, four worked in a school including preschool, three in health care including dental health and the remainder worked in a church, in trading, in a restaurant, in cleaning and in a museum. One male participant was in military service, one was attending an academy, and the other males listed their occupation as in engineering (2 participants), in administration, in care, in construction (3 participants), in assembly, in delivery and in public transport. The study sample had worked between 0.5 and 10.5 years in their current occupation.

**Table 1:** Characteristics of the study sample *N* = 24

	Females (*N* = 12)	Males (*N* = 12)
Age (years)	25–30 (md = 28.5)	26–30 (md = 29.0)
Years in present occupation	0.5–9.0 (md = 4.0)	1.0–10.5 (md = 5.0)

	*N*	*N*
Education		
Nine years of school or high school	6	6
College or university	6	6
Work ability		
Poor (WAS ≤ 5)	3	3
Poor (WAS ≤ 6)	3	3
Excellent (WAS = 10)	6	6

*Md* median, *N* number of workers, *WAS* work ability score

### Data collection

Initially, two pilot interviews were performed using the same procedure as described for the following interviews. The pilot interviews were done by the first author (MB) and followed by discussions with the second author (KH) in order to reflect on and improve the interview technique. Then semi-structured, individual, face-to-face, tape-recorded interviews (Kvale and Brinkmann 2009) were performed by the first author.

All interviews except for one were held at the Department of Occupational and Environmental Medicine in Gothenburg. One interview was done at the participant’s home because of the participant’s concern about the distance to Gothenburg. The interviews were carried out during May–September 2013. An interview guide with fours topics represented by four main questions was used and the interviews lasted between 27 and 66 min (median = 46 min). Each interview began with friendly talk before the recorded interview, to help create a more open and trusting environment, and to increase the possibility of gaining rich descriptions of the participant’s experiences of his or her work ability (Kvale and Brinkmann 2009).

The first question in the interview was about a typical day at work, as the basis was to understand the young adults’ daily work. Then the formal opening question was formulated as followed: *Can you tell me what it means for you to have work ability in your present work?* The other three interview topics concerned experiences of reduced, improved and excellent work ability: *Can you tell me about your experiences when your work ability was decreased? Can you tell me about your experiences when your work ability was increased?* and *Can you tell me about your experiences when your work ability was at its best?* The participants were asked to tell the interviewer about different experiences and give example of occurrences by follow-up questions, for example: Can you give an example? Do you remember an event when you experienced this? As a few of the participants did not bring up any experiences from their personal life, a complementary follow-up question was used: Do you have any experiences outside your working life that have influenced your work ability?

After the interviews, as promised, all participants were given two cinema tickets. The first five interviews were transcribed by the first author and the rest by a secretary, all verbatim. A quality control was performed soon after the transcriptions by having the first author (MB) listen to each interview and compare the recording with the transcribed text (Kvale and Brinkmann 2009).

### Analysis

The analysis followed the guidelines for strategies in systematic text condensation (STC) (Malterud 2012). The data analysis started after the final interview had been held, according to these guidelines. During the analysis process, a bridling of the process of pre-understanding was practiced through reflection and discussions between the two authors (MB, KH) while analyzing the material (Dahlberg et al. 2008). The four recommended steps in STC were followed: (1) preliminary themes connected to the phenomenon of having work ability were emerged. In this first step, the two authors (MB, KH) listened to and carefully read the interviews several times to obtain an overview of the participants’ experiences of work ability. (2) Meaning units related to the phenomenon were identified in each interview and sorted into groups of themes and subthemes, representing different aspects of the participants’ experiences of work ability. This step was performed using the computer program nVivo (Edhlund 2011). In total, approximately 750 meaning units were identified and sorted. (3) The contents in each theme were manually condensed and abstracted into meanings of the phenomenon. In this third step, new terms for the themes and subthemes were also described. (4) The meanings were manually condensed, and descriptions and concepts were developed (Malterud 2012). Thorough the analyses, the number of themes and subthemes were changed several times to end up with four themes, each with three subthemes. The analysis was accomplished by the main author in close collaboration with the second author. Finally, to discuss the process and the results in terms of intelligibility and credibility, a workshop with two colleagues outside the department who had knowledge of phenomenology was held.

### Ethics

The study was approved by the Regional Ethical Review Board at the University of Gothenburg, Sweden, Reg. no. 797-12. To secure the integrity of the participants, written and oral information was given before the interview. This information included the study’s aim and procedure, the issue of confidentiality, the voluntary nature of research and the possibility of withdrawing from participation without explanation. After this, the participants gave their written consent to participate.

## Results

**Fig. 1:**
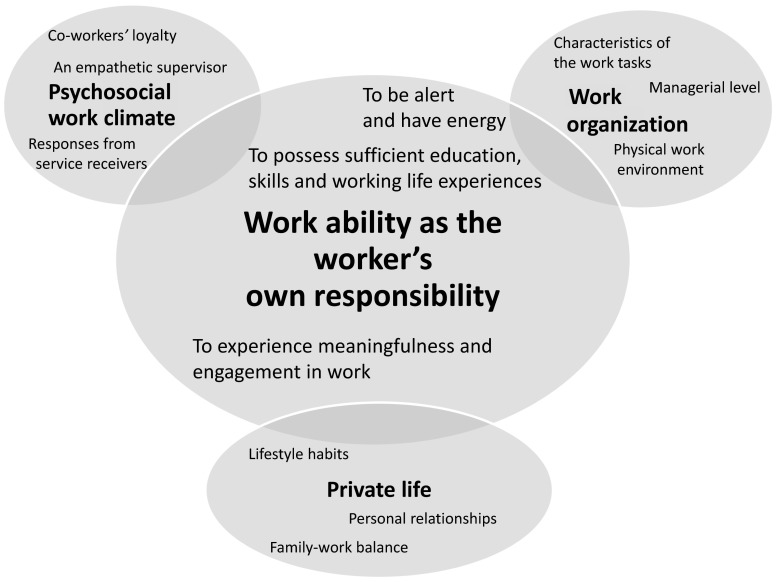
Experiences of work ability among young workers. The participants described work ability as an own responsibility, that can be influenced by the psychosocial work climate, the work organization and the private life. The main themes are in *bolded text* and the subthemes to each main theme are in *plain text*. The core theme is in the *middle*

Young workers experienced work ability as complex, consisting of four themes, each with three subthemes; see Fig. 1. To be alert and have energy and to possess sufficient education, skills and working life experience and experience meaningfulness and engagement in work were perceived to be fundamental and were seen as the worker’s own responsibility. Furthermore, work ability can be improved or reduced by the psychosocial work climate, the work organization and the private life. Optimal work ability was experienced when all themes integrated in a positive way. The participants described that all themes were necessary for the experience of optimal work ability.

### Work ability as the worker’s own responsibility

In this core theme, the participants described what it meant to have work ability. Work ability depended to a great extent on them; they took personal responsibility for it, while they took some parts of work ability for granted. Three subthemes constituted this theme as a whole and were in interplay with each other.

### To be alert and have energy

The participants experienced work ability when they were alert, had energy for the work task, felt well and did not have any sicknesses or injuries. This state of good health was a foundation for having the functions and abilities that were needed in the specific work situation. These functions and abilities were described as the participants’ responsibility in experiencing work ability. Also, they were to some extent taken for granted. When they felt tired and experienced a loss of energy, their work ability was poorer, since they were less effective. However, when they were alert, the physical, psychological (including cognition) and social functions and abilities which were needed to perform the work were intact:

I feel alert and strong, and my back feels strong. I immediately feel much better – I have more energy. I can tell you, it definitely affects your whole day…you feel like a stronger, better human being and then you’re able to deal with everything else. (Care employee, age 28)

### To possess sufficient education, skills and working life experiences

The participants described that sufficient education, skills and working life experiences were needed to experience work ability. Education was seen as a personal requirement and responsibility, to obtain specific work or to be allowed to work in a specific occupation, and education was often taken for granted. More education contributed to better skills and improved working life experience, which led to better work ability. Moreover, the participants described the importance of the professional role. If they failed to live up to this professionalism, their work ability was poorer, but when they experienced more safety and control in the professional role, their work ability was better:

Well, I work with young people, so my work ability is about being able to keep my personal life private. So, like, you can’t … if I’m in a bad mood, I can’t allow it to show. If I am in a good mood, well, I can’t let them notice that either. They’re just so quick to pick up how you’re feeling that you got to be on top of your game all the time, because you work in situations where you are needed and you’ve got to sort of be … my work ability depends on [laughs]… depends on my being in, you know, peak form. (School employee, age 29)

### To experience meaningfulness and engagement in work

The participants described that being motivated, enjoying one’s job, having the will to go to work and be engaged in the work was another basis for experiencing work ability and were described in part as an aspect for which they were personally accountable. When they did well for someone and experienced work as meaningful, their work ability was better. Comfort and joy in the work situation also contributed to good work ability. Furthermore, when the demands from themselves or from the employer were appropriate, their motivation was better, as was their work ability, but when their motivation declined, their work ability was poorer. An own responsibility was described as:

Well, something made me realize that nobody’s going to come and pour a bucket of motivation on me. It’s up to me to be motivated about what I do, and I’ve sort of changed my outlook.” (Master of Science in Engineering, working in industry, age 29)

### Psychosocial work climate

How the participants experienced the psychosocial work climate at the workplace could change their work ability to be better or poorer. A good atmosphere with colleagues was experienced as essential to be able to perform the work well. When the psychosocial work climate was good, their work ability was better. Similarly, the atmosphere with their immediate supervisor could change their work ability, which was better when the participants met appreciation, respect and warmth in this relationship. If these characteristics were absent in their contact with their supervisor, their work ability was poorer. Also, if the participants received positive feedback from the people receiving service from them, the resulting feeling of doing good led to a better work ability. This theme consisted of three subthemes which interacted with each other.

### Co-workers’ loyalty

The participants experienced better work ability when colleagues cooperated well with them and when they had fun together. When the collaboration involved support, it could also reduce the possibility of their work ability being poorer when doing mentally demanding work. A lack of good teamwork, sometimes with conflicts, led to poorer work ability. Good cooperation could also result in new knowledge and skills, through guidance from more experienced colleagues, and consequently better work ability. Receiving appreciation from colleagues, having trust in each other, having good communication and laughing and joking with each other led to a better work ability:

The plumber needs to install connections behind the pipes. If I’m like friends with the plumber, well of course I’ll cut’em and throw’em in. And he will put’em up if I say I don’t have time – got to run – and it’s no problems. But if it was a plumber who was being pig-headed with me, he might have said: “I’m not doing that”. It affects you of course – you get stressed out by stuff like that. It’s much easier if we’re all buddies – all of us pulling in the same direction. It makes such a difference, because everything you do goes so much faster and you feel much better. You have much more fun during your breaks, too. It has an effect, because eventually you lose interest in the work and you don’t produce as much you could. (Construction worker, age 30)

### An empathetic supervisor

The participants experienced better work ability when they received social support, appreciation and encouragement from their immediate supervisor in an interplay characterized by cordiality, trust, helpfulness, understanding and listening. As well, the participants described that a good wage level is a possible form of appreciation. Furthermore, their work ability became better when they perceived the possibility of participation in and influencing their work situation owing to this interplay. In the absence of support and kindness or in the presence of harassment, their motivation was less and their work ability was poorer. When they were met by respect and were treated fairly, their work ability was better:

If my boss sees my strengths and abilities, and challenges me the way she actually sometimes does, then I want to work harder, really focus on certain issues. It’s important for me to feel appreciated. I think it is so for many people. It’s important to feel you’re getting somewhere in your job – not so much career-wise, but knowledge-wise. As a result, my work ability and to focus on my goal becomes greater. (School employee, age 28)

### Responses from service receivers

The participants experienced more meaningfulness and better work ability when they received gratitude and appreciation from customers, patients or pupils. Their work ability was poorer when they met dissatisfaction, but was better when people were content with or expressed delight in the work the participants had performed:

Take the interactions I’ve had with customers. I mean, if I have a friendly or appreciative exchange with someone, I relax. It’s telling me I do a good job. It’s almost like I get a shot of energy from it, and then I do an even better job. (Cleaning staff member, age 29)

### Work organization

The participants described different experiences of work organization as leading to better or poorer work ability. The organizational structure, the attributes of the work tasks and the state of the physical work environment appeared to change their experiences of work ability. This was mainly due to the degree of alertness and meaningfulness, as reflected in the core theme. Mostly, they described negative experiences of poorer work ability when they experienced that the work organization was insufficient. This theme was constituted of three subthemes in close interplay.

### Managerial level

The participants expressed poorer work ability when unclear management practices led to poor communication. This could mean a lack of communication or conflicting instructions. In contrast, the participants experienced better work ability as a result of greater alertness when good scheduling permitted sufficient recovery during their work or in their time off. How specific work tasks were scheduled during the day could also lead to better or poorer work ability. Moreover, they described that work ability decreased when organizational changes such as down-sizing or a lack of stand-ins occurred, especially when the nearest leader lacked decision-making in the situation:

She (the supervisor) … you know, I’m really of two minds: I know she has demands on her from above – she has to get certain things done. She says, okay, we’re taking in these children, so … make it work! And it takes time, too, figuring out where we’re going to put all these children. It takes time when a staff-member has to leave the room so she can participate in a planning group. If we don’t get a replacement for that person, there’s more pressure on me, and more stress. It means I can’t just go and be with the group of children when I want to. It becomes this kind of carousel that spins round and round really fast. Everything ends up being negative, and that affects me and my work ability. (Pre-school employee, age 29)

### Characteristics of the work tasks

The participants commented that they experienced greater efficiency and work ability when routines and order at the workplace were straightforward. Too many work tasks at the same time or insufficient time for work tasks led to less work ability, compared with better work ability when they had an appropriate number of work tasks for a day or a specific period. Repetitive or boring work tasks gave rise to poorer work ability and a possible feeling of being replaceable. Work ability became better when the participants experienced fun, challenging, stimulating and varying work tasks:

For example, when I’m at somebody’s home, installing a fan in, say, a kitchen – that’s fun work. You put in a little extra effort so it will look great, not merely work. Like how you clean up after yourself and things like that … and do as good a job as you can. Of course it affects your work ability – no doubt about it. (Construction worker, age 29)

### Physical work environment

The participants described work ability as being better or poorer owing to the physical work environment, including physical demands. Poorer work ability because of pain was described when frequent lifting and unfavorable working postures due to insufficient workplace design were common. Working indoors, poor ventilation, inadequate heating and limited working space could lower work ability. Outdoor work with different weather, light and temperature could change work ability also, with rain, excessive heat or excessive cold tending to decrease it:

I try to make sure I wear long-johns, try to stay warm. But I definitely feel the cold. … it’s mainly in the terminal – like when we’re standing inside sorting and then drive out the packages. We have to take our outer clothes off so they won’t get in our way, and then we really feel it. And that’s when we’re still inside, before we even started to drive. Your movements get slower, and yes, you get pain. Then there’s a risk the next day will be even worse if you don’t treat it. Then again, many people think it doesn’t really matter – and then you just get stiffer and stiffer and stiffer. So I would actually say, yes, absolutely, it does affect your work ability. (Delivery industry employee, age 26)

### Private life

The participants’ life outside work could alter their experiences of work ability. Lifestyle habits such as sleep, diet and physical activity, the quality of their personal relationships and the balance between private life and work could increase or decrease their work ability. If the participants practiced good routines in sleeping, eating and being physically active, they were more alert and experienced better work ability. Good personal relationships supported better work ability in contrast to poor personal relationships or life crises. Also, it appeared that a balance between private life and work was essential in brought possibilities to recovery. Three subthemes together formed this theme.

### Lifestyle habits

The participants emphasized that sleep was needed to remain alert, which then supported better work ability. Good sleep gave a feeling of being thoroughly rested, which led to better work ability; even one single night with less sleep could result in poorer work ability. Also, if they ate on time and performed physical activity regularly, their work ability was better. The participants described that decreased physical activity during a period resulted in poorer work ability, especially in physically demanding work, as they became more tired. Experiences of lack of sleep in relation to work ability were described as:

And it’s easier to make mistakes when you haven’t had enough sleep, you can say. Then, and this is the worst thing of all, the worst is that you could lose your concentration when driving. Worst-case scenario – luckily it hasn’t happened to me, nor to anyone I know, as far as I know– but the worst-case scenario is that you could fall asleep at the wheel. That is the worst conceivable outcome I can see from this. So no doubt about it, not getting enough sleep reduces your work ability. (Public transit employee, age 29)

### Personal relationships

The participants described that when they had good relationships with family and friends, their work ability was better. On the other hand, life crises such as sickness or death in the family and conflicts with family members led to poorer work ability:

Well, the thing is, if you’re having problems at home, it will affect how you perform … at work, maybe, at least these other tasks that don’t involve patients –administrative tasks. You might not … if you’re not feeling well, you’re not able to get as much done. (Health care employee, age 29)

### Family–work balance

The participants viewed the balance between family life and work as a source of recovery and better work ability. If they experienced an imbalance in life because of problems outside work, their work ability was poorer. To separate work and private life could in some cases favor better work ability, and a family life with rich leisure time could also be positive:

I also believe it’s important to be able to take a break from your work, because if you don’t recharge your batteries by doing things you enjoy doing just for the fun of it, whatever recreational activity it might be, you won’t have the stamina or the energy to do your work. (Church employee, age 27)

## Discussion

The young workers experienced work ability as an own responsibility (the core theme) that could be influenced by their psychosocial work climate, work organization and private life. Each of these four themes included three subthemes, describing a complex picture of the participants’ experiences of their work ability. When the core theme was optimal and was influenced by all the other three main themes in a positive way, optimal work ability was experienced. To our knowledge, these findings for young workers have not earlier been shown.

Concerning the finding of individual responsibility the participants did not view their work ability as being dependent on the organizational structure of their workplace. The participants stated it was up to them to be alert and have energy so they could use their functions and abilities, to acquire education, skills and working life experiences, and to perceive meaningfulness and engagement in work. An increased individualism in the younger generation could be one explanation (Gillberg 2010). In his thesis of young adults’ perceptions of work and self-fulfillment, Gillberg highlighted a change in their working life, in comparison with earlier generations of workers where the goals and results of work had been an employer responsibility. Today, more people are forced to plan, perform and take responsibility for their actions at work. Likewise, the increased individualism contributes to a larger responsibility for young people to plan their life path on their own (Beck and Beck-Gernsheim 2005).

Experiencing work ability as a personal concern could also be attributable to young workers being unaware of their employers’ responsibility for the work environment, possibly resulting from their inexperience, a factor that has been discussed in earlier studies (Kines et al. 2013). In the present study, the participants’ limited experience of working life could to some extent be another explanation for their focus on their personal responsibility. The participants had between 0.5 and 10.5 years of experience in their current occupation, which might be too short a time to become aware of the possible influence of their work organization on their work ability.

Another finding in the present study was the participants’ experiences of the complexity of their work ability. To experience optimal work ability, the importance of all themes interacting was distinct. These results can be a complement to the Finnish model, the house of work ability. In this model, work ability is described as the balance between individual resources and work demands (Ilmarinen 2009). The house of work ability has four levels, where the first level consists of health and functional capacity, the second of knowledge and skills, the third of values, attitudes and motivation, and the fourth level of different aspects of work. Work ability is the roof of the house and symbolizes this balance, in close interaction with the private life and the society outside the house.

The entire first theme in the current study was experienced as a core and a foundation for work ability, in some way unlike to the most important first level of health and functional capacity in the house of work ability (Gould et al. 2008; Ludvigsson et al. 2006). Also, in the house of work ability, knowledge and skills, and values, attitudes and motivation have been found to be less significant for work ability than the first and fourth levels of health and functional capacity, and different aspects of work (Ilmarinen et al. 2005). The findings in the current study showed a slightly different picture. Finally, the participants in the present study included their private life in their experiences of work ability, somewhat unlike the house of work ability where private life is not a part of the house, although it is in a close interaction. In total, the found themes in the current study are slightly different from the themes in the house of work ability and seem to have dissimilar weightings. Nevertheless, these results may be valid for other age groups as well, but comparative explorative studies in future research of adult workers would be needed to answer this.

In the core theme the characteristic to be alert and have energy was described in terms of feeling well and not having any illness or injuries and was a basic condition for work ability. This finding is in line with previous Finnish quantitative studies of work ability among young workers (Gould et al. 2008; Pohjonen 2001), where perceived poor health was shown to most clearly relate to poor work ability. Furthermore, a lack of health, with chronic symptoms in the upper extremities and widespread symptoms in the neck and upper extremities, has also been shown to relate to reduced productivity among young students (Bostrom et al. 2008). Although the concept of reduced productivity is not exactly the same as poor work ability, it is closely related (van den Berg et al. 2011).

To be alert and have energy was also a requirement of having different personal functions and abilities—physical, psychological (including cognition) and social—necessary to experience work ability. This findings is in line with the results of one review (Fadyl et al. 2010), where physical and psychological function were found to contribute to work ability among injured adult workers. As well, the findings in the current study are in agreement with the results of another review (van den Berg et al. 2009), whose authors reported that poor musculoskeletal capacity was associated with poor work ability in adult workers.

Sufficient education, skills and working life experiences were also found to be a foundation for work ability in the present study. Guidance by experienced co-workers was mentioned as improving skills and accordingly work ability, and this guidance has also been shown to be important among young construction workers (Holte and Kjestveit 2012). In the previously mentioned review of injured adult workers (Fadyl et al. 2010), the authors stated that thinking and problem-solving skills as well as social and behavioral skills contributed to better work ability among these workers; the same results were seen in the present study.

The last characteristic in this first theme addressed the young workers’ experiences of meaningfulness and engagement in work. Meaningfulness has earlier been shown to have a positive relationship to engagement (May et al. 2004) and meaningful work has been emphasized among adults (Cartwright and Holmes 2006; Hasselkus 2002). To have fun at work increased the motivation, the participants stated an attitude previously confirmed among young workers (Loughlin and Barling 2001). Also, commitment and interest have been highlighted among employers as a positive aspect of their employees’ work ability (Jansson 2014). Finally, experiencing work as meaningful appears to be just as important to the younger generation as to the previous generation, as indicated in a recent review (Twenge 2010).

For the other three main themes: psychosocial work climate, work organization and private life, several findings were in line with earlier research (Fischer et al. 2005; Gould et al. 2008; Pohjonen 2001; van den Berg et al. 2009) on mainly adult workers, as research on young workers are more scarce.

### Methodological consideration

Qualitative research can both broaden and deepen medical knowledge and understanding of humans (Malterud 2001). In this study, alternative criteria for validity and reliability in a qualitative study were used: creditability and dependability (internal validity), conformability (objectivity) and transferability (generalizability) (Lincoln and Guba 1985). These aspects are illustrated below in different ways. Furthermore, standards for reporting qualitative research (Tong et al. 2007) have been followed in this paper, in agreement with the selected strategy.

The structural work process with a phenomenological approach that was used in this study was based on the work of Malterud (Malterud 2012). The use of a few open questions made it possible to access each participant’s experiences more deeply and to gain rich data. Also, the foundation in phenomenology in performing research with an open mind was helpful to obtaining useful material. Nearly all of the participants could relate to the concept work ability and describe experiences of it in their daily work. However, two of the participants needed the first question to be paraphrased as “What is required to have work ability in your daily work?” which might be interpreted in a more personal way.

A careful selection of the study sample was performed. Through this strategy and also through the randomization in the subgroups, a large variation in experiences could be obtained, which is crucial for the quality of this type of study (Hallberg 2013). The selection of participants with different education levels, which contributed to a large variation of occupations, can be seen as a strength in this study. This facilitates a possible transferability of the results to similar contexts with young adults, 25–30 years old, in other areas of Sweden (Hallberg 2013; Malterud 2001). Also, the variation in self-reported work ability provided different experiences in work ability related both to well-being and to unhealthy conditions such as depression and musculoskeletal disorders.

One limitation in this study is the overrepresentation of young adults born in Sweden, both in the study sample and in the original cohort (Ekman et al. 2008). Consequently, the results would possibly not be transferable to areas in Sweden that are home to many young people born outside Sweden or with parents born elsewhere.

The individual interview method was used as the aim was to explore individual experiences of work ability in depth. This method is in contrast to focus groups methodology, which is often used to give a collective understanding of the world (Ivanoff and Hultberg 2006). The selected strategy, systematic text condensation (STC) was a pragmatic choice of a transparent, systematic and easy-to-use procedure of analysis (Malterud 2012). A clear description of the steps in the analysis facilitates not only the procedure for the researcher, but also the understanding and the assessment of dependability and credibility for the reader (Malterud 2001). The strategy of presenting a few open research topics increased the possibility of finding something new and interesting in the study (Kvale and Brinkmann 2009). The workshop at the end of the process also contributed to an increase in the quality of the study, specifically the study’s specific aim, the qualitative language, the philosophy base, the quotations and the understanding of the results.

Reflection on how the researchers can influence the research process is necessary throughout the process (Tong et al. 2007). The bridling of the preconceptions and pre-understanding during the interviews gave an attitude of not knowing the answer in advance through the use of open questions and follow-up questions (Dahlberg et al. 2008). After both the pilot interviews and the actual interviews, feedback on the first author’s actions was discussed with the second experienced researcher, which can be seen as a validation of the study’s ability to yield rich and relevant data. Although work ability has been explored in other qualitative studies (Jansson 2014; Stigmar et al. 2010, 2012), the present study is the first, to our knowledge, to qualitatively explore work ability among a group of young workers.

### Applications

The results in this study can increase knowledge about young workers’ experiences of work ability. Their experiences of their personal responsibility for work ability and the complexity of work ability could be useful in promoting good work ability for this group. Perhaps the young workers’ limited awareness of the importance of a good work organization for improved work ability can be highlighted for employers, as the responsibility for good work ability has to be shared between the employer and the employee (Ilmarinen 2009). Hence, a well-performed introduction and education of new employed workers could be of great importance.

Employers’ knowledge of the importance of a balance between private life and work for the employees can be a key in helping employees to stay alert and have energy. Furthermore, creating meaningful work where people are engaged and motivated and also have sufficient knowledge and skills is essential for work ability. In future research, explorative interview studies of work ability in different age groups could clarify similarities or differences between young and older workers.

## Conclusions

Work ability was experienced as the worker’s own responsibility that could be influenced by work circumstances and private life. To promote good work ability among young workers, work ability has to be understood in its specific context. Whether the understanding of work ability found in this study is explicit for the group of young adults needs to be explored in a more general population in further research.
